# Combined Effects of Irradiation, Nutrients, and Cyanobacterial Composition on Microcystin Concentration in Chinese Plateau Lakes

**DOI:** 10.1021/envhealth.5c00123

**Published:** 2025-07-10

**Authors:** Hexiang Zhao, Xu Zhao, Ying Liu, Yanfeng Sun, Youai Duan, Jun Chen, Ping Xie, Yong Liu, Xinyu Miao, Haijun Wang, Chi Xu, Qian Liu, Wenyu Yang, Christian E. W. Steinberg, Hugh MacIsaac, Susanna A. Wood, Erik Jeppesen, Hans Paerl

**Affiliations:** † Yunnan Key Laboratory of Ecological Protection and Resource Utilization of River-lake Networks, Qilu Lake Field Scientific Observation and Research Station for Plateau Shallow Lake in Yunnan Province, Institute for Ecological Research and Pollution Control of Plateau Lakes, School of Ecology and Environmental Science, 12635 Yunnan University, Kunming 650500, China; ‡ Donghu Experimental Station of Lake Ecosystems, State Key Laboratory of Freshwater Ecology and Biotechnology, 53021 Institute of Hydrobiology, Chinese Academy of Sciences, Wuhan 430072, China; § School of Life Sciences, 12581 Nanjing University, Nanjing 210023, China; ∥ State Key Laboratory of Environmental Chemistry and Ecotoxicology, 26442 Research Center for Eco-Environmental Sciences, Chinese Academy of Sciences, Beijing 100085, China; ⊥ Institute of Biology, Freshwater & Stress Ecology, Humboldt University, Berlin 12437, Germany; # Yunnan Provincial Key Laboratory of Soil Carbon Sequestration and Pollution Control, Faculty of Environmental Science & Engineering, Kunming University of Science & Technology, Kunming 650500, China; ∇ 2244 Lincoln University, 85084 Ellesmere Junction Road, Lincoln 7647, New Zealand; ○ Great Lakes Institute for Environmental Research, 8637 University of Windsor, Windsor, Ontario N9B 3P4, Canada; ◆ Department of Ecoscience, 1006 Aarhus University, Aarhus 8200, Denmark; ¶ Sino-Danish Centre for Education and Research, Beijing 101408, China; †† Institute of Marine Sciences, Department of Earth, Marine and Environmental Sciences, University of North Carolina at Chapel Hill, 3431 Arendell Street, Morehead City, North Carolina 28557, United States; ‡‡ Yunnan Institute of Water & Hydropower Engineering Investigation, Design and Research, Kunming 650032, China; §§ Yunnan Ecological and Environmental Monitoring Center, Kunming 650034, China

**Keywords:** Yunnan Plateau, microcystin, cyanobacteria, nutrients, solar radiation intensity

## Abstract

Microcystins (MCs) are one of the most prevalent cyanotoxins and pose significant risks to aquatic ecosystems and human health, particularly in lakes used as drinking water sources. However, knowledge about the MC concentrations in plateau lakes experiencing high solar radiation is scarce. This study investigated the spatial-temporal distribution of MCs in eight Yunnan Plateau lakes in China, focusing on their relationships with environmental factors. Water samples (*n* = 63) were collected during summer and winter seasons and analyzed for MC concentrations along with a suite of environmental variables. Results revealed significant seasonal and spatial variations in MC concentrations, with higher levels in eutrophic lakes Dianchi, Erhai, and Xingyunhu. Notably, mean MC concentrations in Lake Dianchi during summer and Erhai during winter exceeded the World Health Organization’s provisional guideline of 1 μg/L for drinking water. Seasonal analyses revealed distinct regulatory mechanisms: MC concentrations in summer were positively correlated with total phosphorus, total nitrogen, turbidity, and chlorophyll *a*, reflecting the influence of eutrophication on cyanobacterial growth. While solar radiation intensity (SRI) exhibited a dual role: moderate SRI in winter was associated with higher MC levels, whereas higher SRI in summer suppressed MC production, likely due to photoinhibition or MC degradation. Strikingly, water temperature showed no significant correlation with MC concentrations, suggesting that high solar radiation in the Yunnan Plateau may override temperature-dependent effects on cyanobacterial growth. These findings highlight the importance of nutrient management and the regulatory role of solar radiation in regulating MC production in high-altitude lakes. The study underscores the need for region-specific strategies to mitigate cyanobacterial risks, particularly in drinking water source lakes, by integrating nutrient control and the unique light regime of plateau ecosystems.

## Graphical abstract

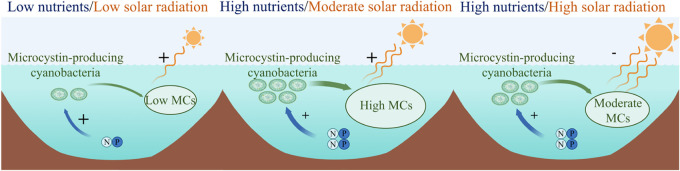

## 1 Introduction

Cyanobacterial toxins, as secondary metabolites, constitute a significant threat to aquatic ecosystems, wildlife, and human health.^1^ Among these toxins, microcystins (MCs), a class of cyclic heptapeptide hepatotoxins, are among the most prevalent cyanobacterial toxins globally.^2–4^ The increasing frequency of cyanobacterial blooms, driven by anthropogenic nutrient inputs and climate change, has exacerbated the risk of these toxins.^5,6^ These toxic cyanobacterial blooms not only threat aquatic ecosystems but also endanger humans and wildlife who might be exposed through direct contact, ingestion, or inhalation.^7–10^ Beyond their well-documented hepatotoxic effects, MCs have recently exhibited potent neurotoxicity in various animal models.^11–15^

Notable negative impacts on wildlife include the mass mortality of lesser flamingos (*Phoenicopterus minor*) in East Africa in 1999,^16,17^ the death of bald eagles in the United States due to cyanobacterial-induced vesicular myelin disease,^18^ and the 2020 mortality of 356 African savannah elephants in Botswana attributed to MC ingestion.^19,20^

Environmental factors influencing MC production and toxicity have been extensively studied through field surveys^21–23^ and laboratory settings.^24–29^ Key factors such as temperature, light intensity, and nutrient (nitrogen and phosphorus) concentrations have been shown to regulate MC production.^30^ However, their effects can vary significantly between lowland and plateau lakes due to differences in elevation, temperature, and solar radiation. For instance, Yunnan Plateau lakes, is characterized by lower mean annual temperatures (15 °C) and higher solar radiation intensity (207 W/m^2^) compared to lowland regions like the Pearl River Delta (24 °C and 191 W/m^2^), creating unique conditions for MC generation.^31^ Previous studies have shown that MC production by *Microcystis* and *Dolichospermum* (previously *Anabaena*) peaks at 18–25 °C,^32^ while high light intensities can suppress MC production and promotes photodegradation with potential toxic byproducts, further complicating their environmental impact.^33,34^

Geographic and climatic differences between lowland and plateau regions also influence eutrophication patterns and nutrient dynamics in lakes, which in turn affect MC production.^35^ For example, high nitrate concentrations promote cyanobacterial growth and MC production,^36,37^ while the regulatory role of the nitrogen control gene *ntcA* adds complexity to MC production under varying environmental conditions.^30^ Notably, most studies have focused on lowland waterbodies or controlled laboratory experiments, leaving a critical knowledge gap regarding MC dynamics in plateau lakes. A recent Web of Science search (March 23, 2025) revealed studies focusing on Yunnan Plateau lakes accounted for merely 0.18% (6/3,260) of cyanotoxin publications, highlighting the need for more region-specific research. Given the unique environmental conditions of plateau lakes, such as higher solar radiation and lower temperatures, we hypothesize that MC concentrations in Yunnan Plateau lakes are jointly regulated by a combination of eutrophication status and light intensity. To test this, we conducted a comprehensive survey across eight Yunnan Plateau lakes with varying degrees of eutrophication levels during both summer and winter seasons. This study aims to elucidate key drivers of cyanobacterial abundance and MC concentrations in plateau lakes, providing valuable insights for the management and mitigation of cyanobacterial blooms in these unique ecosystems.

## 2 Materials and Methods

### 2.1 Study Area

The Yunnan Plateau ranges from a high altitude in the northwest to a lower altitude in the southeast, with an average elevation of approximately 2000 m (from 76.4 to 6740 m). Dominated by a subtropical monsoonal climate, the region features minimal annual temperature differences but distinct dry and wet seasons during the year. Over 40 lakes exist on the plateau, most of which are tectonic fault lakes differing in trophic state and anthropogenic pressure. This study focused on eight lakes in the Yunnan Plateau: Luguhu, Chenghai, Yangzonghai, Erhai, Xingyunhu, Dianchi, Yilonghu, and Qiluhu. These lakes span a gradient from oligotrophic to eutrophic, with surface areas ranging from 27 to 298 km^2^ and maxium depth from 6.6 to 93.5 m. Five lakes (Luguhu, Chenghai, Yangzonghai, Erhai, and Dianchi) serve as direct or alternate drinking water sources, while the remaining three (Xingyunhu, Yilonghu, and Qiluhu) are used for recreational activities (Table S1), reflecting their varying human impact and ecological sensitivity.

### 2.2 Field Sampling and Lab Analysis

Near-surface water (0.5 m depth) was collected from the plateau lakes in summer (wet season, July 1–21, 2022) and winter (dry season, December 16, 2021–January 3, 2022) to analyze the concentration of MCs, physicochemical parameters, and phytoplankton community structure. We examined 63 sampling sites in eight lakes (Figure 1b). Surface water was collected using a Plexiglas sampler (JC-800, 5 L) and stored in 1 L brown polypropylene (PP) bottles for MCs and 1 L white PP bottles for water quality. The samples were stored at 4 °C and returned to the laboratory for analysis. For phytoplankton quantification, we collected 5 L water samples using the same Plexiglas sampler, stored them in opaque white PP reagent bottles, and fixed on site with Lugol’s Iodine. In the laboratory, phytoplankton samples were allowed to settle for 48 h, after which the supernatant was siphoned off, and the remaining sample was concentrated to 30–50 mL for microscopic identification and enumeration.

**Figure 1:**
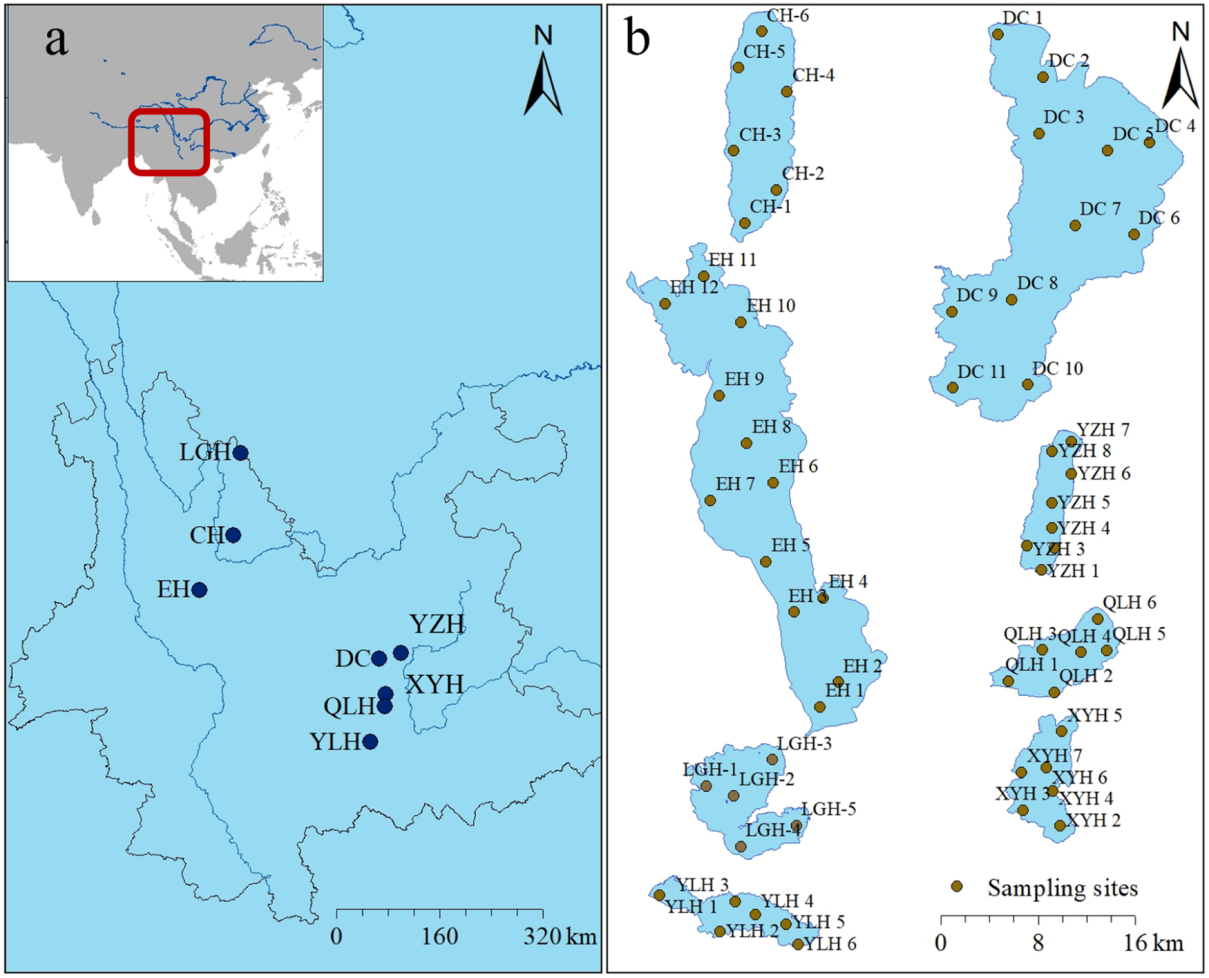
Location of the eight studied lakes in Yunnan Plateau, China (a) and the distribution of the sampling sites; (b) for each lake. LGH, Lake Luguhu; CH, Lake Chenghai; YZH, Lake Yangzonghai; EH, Lake Erhai; XYH, Lake Xingyunhu; DC, Lake Dianchi; YLH, Lake Yilonghu; QLH, Lake Qiluhu.

Field-collected water samples were vacuum filtered through a glass fiber membrane filter (Whatman GF/C, UK) to separate intracellular MCs from extracellular MCs. Extracellular MCs in the filtrate were enriched using a solid-phase extraction column (HLB cartridges, 200 mg, Oasis, Waters, Milford, MA). First, the columns were washed with methanol (5% v/v, 15 mL), then eluted with 10 mL methanol. The eluate was dried at 40 °C under nitrogen gas and resuspended in 1 mL of methanol. For intracellular MCs, GF/C filters were placed in methanol extraction (75%, 30 mL), shaken at 4 °C for 0.5 h at 2000*g*, and centrifuged at 2000*g* for 0.5 h. The supernatant was diluted 6-fold to a volume of 180 mL. MCs were determined using ultraperformance liquid chromatography (UPLC, H-Class, Waters) coupled with a triple quadrupole mass spectrometer (XEVO-TQD, Waters). The three MC congeners (MC-LR, MC-RR, MC-YR) were calibrated (*R* ^2^ ≥ 0.99) over a range of 0–200 μg/L. Quantification was performed by the external standard method, and data quality was ensured by measuring blanks and standards after every 10 samples. MC-LR, MC-RR, and MC-YR standards were obtained from Sigma-Aldrich (≥95%, Munich, Germany).

Physical-chemical factors including water temperature (WT), dissolved oxygen (DO), pH and conductivity (Cond) were measured in situ using a multiprobe water quality sonde (Yellow Spring Instruments, YSI 6600). Turbidity (Turb) was measured with a turbidimeter (HACH 2100Q), water depth (*Z*_M_) with a portable depth sounder (Speedtech SM-5), and water clarity (*Z*_SD_) with a Secchi disk. TN was determined using the alkaline potassium peroxymonosulfate digestion spectrophotometer method (HJ 636-2012), TP using the potassium peroxymonosulfate digestion - molybdenum antimony spectrophotometric method and phytoplankton chlorophyll *a* (Chl *a*) by spectrophotometry (SL 88-2012).^38^ The identification of planktonic algal species was based on the “The freshwater algae of China: systematics, taxonomy and ecology”.^39^ Known MC producing genera present in the lakes were combined into density and biomass of microcystin-producing cyanobacteria (DCyan-MP and BCyan-MP), including *Microcystis* sp., *Planktothrix* sp., *Dolichospermum* sp., *Oscillatoria* sp., and *Pseudoanabaena* sp., and their percentage (DCyan-MP% and BCyan-MP%).

Solar radiation intensity (SRI) and air temperature (AT) data on the eight lakes were derived from the data set “ERA5 Single Levels Monthly Means from 1940 to Present” (https://cds.climate.copernicus.eu) in October 2023. SRI represents net shortwave radiation (incident minus reflected) at the Earth’s surface, derived from hourly accumulated values. We compared the collected solar radiation data with publicly available meteorological data at the sampling time and location to ensure the authenticity and availability of the data.

### 2.3 Data Processing and Analyses

We systematically analyzed the data to identify key drivers of cyanobacterial composition and microcystin (MC) concentrations. Prior to conducting Random Forest model analysis, data below the detection limit was excluded to ensure data quality. The initial variable selection was based on significant Spearman correlations (*P* < 0.05), which were subsequently incorporated into the model. All variables underwent log-transformation before linear regression analysis to meet statistical assumptions. For environmental processing we analyzed solar radiation intensity and temperature data using the tabular display of the zonal statistics program in ArcMap 10.8. The interaction of air temperature and solar radiation intensity (AT*SRI) was quantified by calculating their product representing their combined environmental effect. Statistical analyses were performed using multiple approaches: Spearman rank correlation analyses were conducted with the ggcor package in R 4.2.2, while multiple stepwise regression analysis were carried out using IBM SPSS Statistics 27 software. We generated all sampling location map using ArcMap 10.8 to visualize spatial distributions. Random Forest model analysis was implemented through the linkET package in R 4.2.2, with variable importance assessed via permutation tests. To characterize direct and indirect relationships, a partial least-squares structural equation model (PLS-SEM) was constructed in R4.2.2 using the plspm package, incorporating AT*SRI, nutrients (total nitrogen and total phosphorus), algae abundance (including Chl *a*, DCyan, BCyan, DCyan-MP, and BCyan-MP), and Cyan-MP% (including DCyan-MP% and BCyan-MP%). This model was grounded in ecological theory, where nutrients drive algal growth, subsequently regulating MC production through phytoplankton metabolic processes that are further modulated by light-temperature interactions. Though the data from the field survey in summer and winter are time-limited, they are a reflection of fast-growing season and low-growth season for phytoplankton. Given the non-normal distribution of our data set, we employed nonparametric statistical methods, specifically selecting the Mann–Whitney U test implemented in Origin 2021 to evaluate seasonal variations in physicochemical factors, phytoplankton factors and MC concentrations. While multiple sampling stations per lake might raise concerns about pseudoreplication and inflated statistical power, the considerable distances between stations (>100 m) guaranteed their independence in our study. MC-RR and MC-YR were excluded from various correlation and regression analyses because of their low concentrations and contributions to the total concentrations, and also because of their relative weak toxicity compared to MC-LR.^1^

Ecological risk assessment in this study is based on the optimized risk quotient (RQ_f_), which is calculated using the following equation^40^

1 $${\mathrm{RQ}}_{f}=\mathrm{RQ}\times F=\left(\frac{\mathrm{MEC}}{\mathrm{PNEC}}\right)\times F$$

2 $$F={\mathrm{NO}}_{1}/{\mathrm{NO}}_{2}$$

where RQ is the risk quotient, MEC is the measured concentration of MCs, PNEC is the predicted no-effect concentration of MCs (5.73 μg/L),^41,42^ *F* is the frequency with which MEC exceeds PNEC, NO_1_ is the number of samples above PNEC, and NO_2_ is the total number of samples. RQ_f_ is classified into five risk levels: high risk (RQ_f_ ≥ 1), moderate risk (1 > RQ_f_ ≥ 0.1), tolerable risk (0.1 > RQ_f_ ≥ 0.01), negligible risk (0.01 > RQ_f_ > 0), and safe (RQ_f_ = 0).

## 3 Results

### 3.1 Physicochemical Properties, Phytoplankton, and Microcystin Concentrations

Nutrient levels and phytoplankton status exhibited significant variations among lakes and seasons (Figures 2, 3 and Tables S2, S3). Turbidity in the eight lakes averaged 18.8 NTU (range: 0.3–101.0 NTU) in summer and 18.2 NTU (range: 0.3–57.7 NTU) in winter. Total nitrogen (TN) showed means of 1.64 mg/L (range: 0.08–12.26 mg/L) in summer and 1.52 mg/L (range: 0.11–3.95 mg/L) in winter, with marked seasonal differences observed only in Lake Chenghai and Lake Erhai. Total phosphorus (TP) levels averaged 0.06 mg/L (range: 0.003–0.15 mg/L) in summer compared to 0.08 mg/L (range: 0.001–0.19 mg/L) in winter, maintaining seasonal consistency in Lake Luguhu and Lake Qiluhu. Chlorophyll *a* (Chl *a*) averaged 40.5 μg/L (range: 0.5–176.3 μg/L) in summer versus 24.9 μg/L (range: 0.6–83.3 μg/L) in winter, with Lakes Luguhu and Yangzonghai demonstrating particularly stable interseasonal concentrations. Cyanobacterial cell density (DCyan) showed distinct seasonal patterns, averaging 5.5 × 10^8^ cells/L (range: 2.0 × 10^4^–4.4 × 10^9^ cells/L) in summer and 1.2 × 10^8^ cells/L (range: 1.5 × 10^3^–1.1 × 10^9^ cells/L) in winter. Cyanobacterial biomass (BCyan) reached 18.7 mg/L (range: 0.001–114.9 mg/L) in summer and 4.6 mg/L (range: 1.1 × 10^–4^–42.3 mg/L) in winter. Among the five major microcystin-producing genera (BCyan-MP), including *Microcystis* sp., *Dolichospermum* sp., *Pseudoanabaena* sp., *Planktothrix* sp., and *Oscillatoria* sp., Lake Qiluhu consistently showed the highest biomass values during both sampling periods.

**Figure 2:**
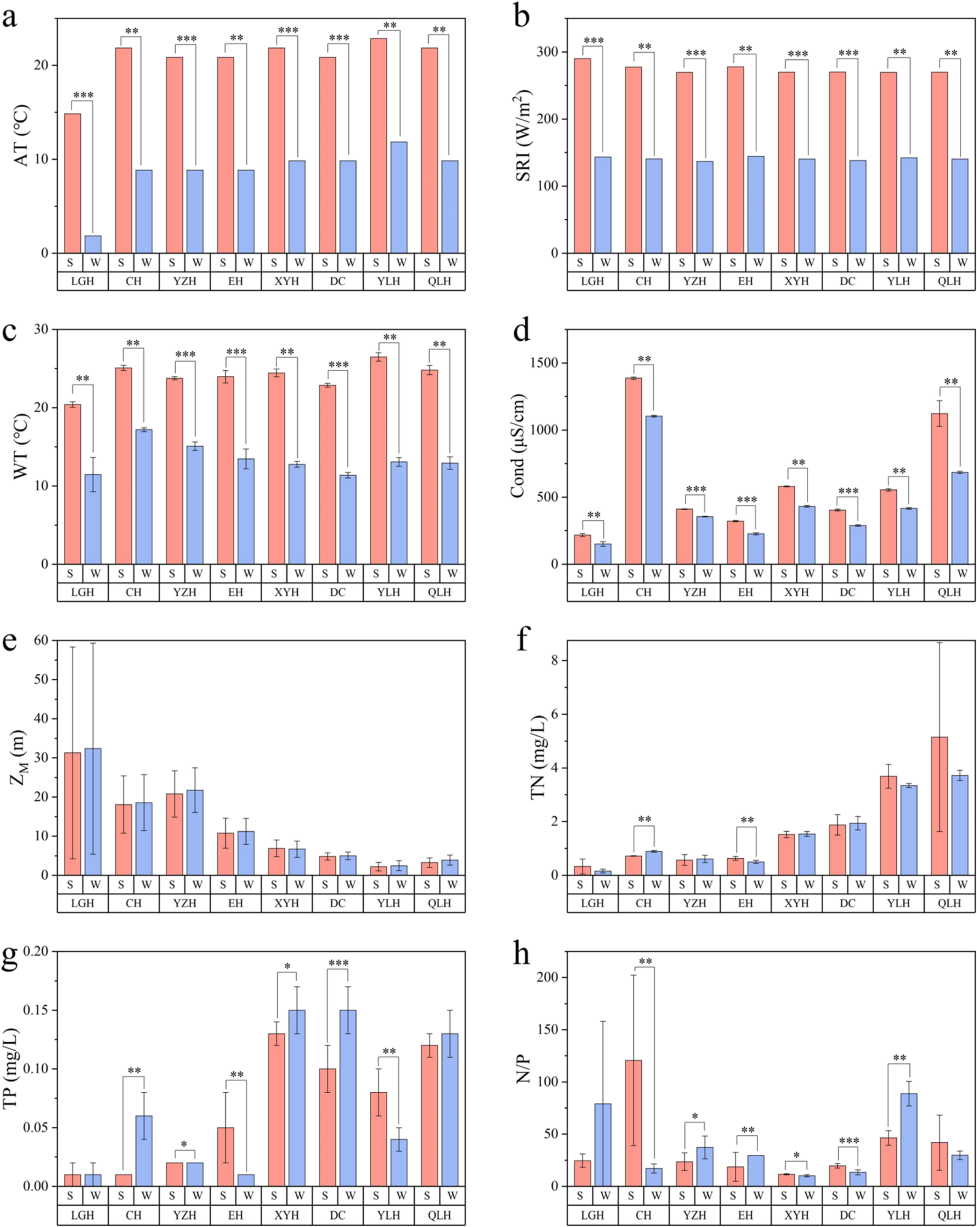
Main physicochemical factors (mean ± s.d.) of the eight studied lakes. (a) AT, air temperature; (b) SRI, solar radiation intensity; (c) WT, water temperature; (d) Cond, conductivity; (e) *Z*_M_, mean depth; (f) TN, total nitrogen; (g) TP, total phosphorus; (h) N/P, ratio of total nitrogen to total phosphorus. Asterisks (*) indicate significant seasonal differences based on nonparametric tests, *P ≤ 0.05, **P ≤ 0.01, ***P ≤ 0.001. Refer to Figure 1 for lake abbreviations. S = summer (red); W = winter (blue).

**Figure 3:**
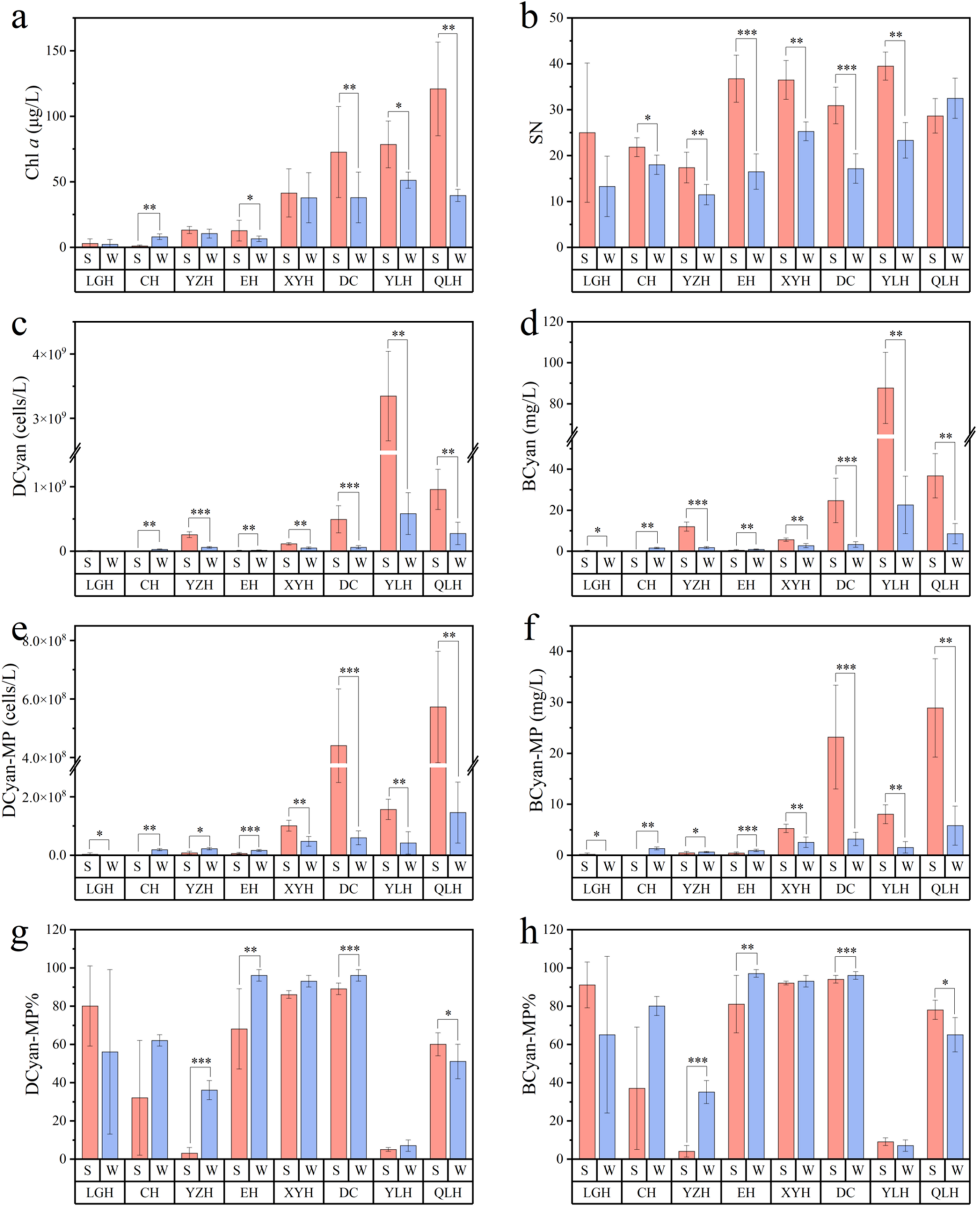
Main phytoplankton metrics (mean ± s.d.) in the eight studied lakes. (a) Chl *a*, phytoplankton chlorophyll a; (b) SN, species number; (c) DCyan, cyanobacteria cell density; (d) BCyan, cyanobacteria biomass; (e, f) DCyan-MP (BCyan-MP), density (biomass) of MC-producing cyanobacteria; (g, h) D (B) Cyan-MP %, percentage of D (B) Cyan-MP to D (B) Cyan. Asterisks (*) indicate significant seasonal differences based on nonparametric tests, *P ≤ 0.05, **P ≤ 0.01, ***P ≤ 0.001. Refer to Figure 1 for lake abbreviations. S = summer (red); W = winter (blue).

The total concentration of MCs in the eight lakes averaged 0.29 μg/L (median: 0.057 μg/L; range: 5.7 × 10^–4^–1.9 μg/L) in summer and 1.32 μg/L (median: 0.058 μg/L; range: 0.001–22.1 μg/L) in winter. Significant seasonal differences in MC concentrations were observed in lakes Chenghai, Yangzonghai, Erhai, Dianchi, and Qiluhu, with higher winter concentrations in Lake Chenghai and Lake Erhai. Among the five lakes serving as direct or alternate drinking water sources, 15% of the sampling sites (13 out of 84) exceeded the World Health Organization (WHO) guideline of 1 μg/L for drinking water. In contrast, none of the 37 sampling sites in the three recreational lakes exceeded the WHO’s provisional guideline of 24 μg/L.

The compositional analysis indicated that intracellular MCs accounted for 89% + _ 13% (mean + _ SD) of total MCs, while extracellular MCs accounted for the remaining 11% + _ 13%. Among the three major MC variants, MC-LR was most prevalent (49% + _ 18%), followed by MC-RR (36% + _ 21%) and MC-YR (15% + _ 14%). The study revealed Lake Dianchi had the highest MC concentrations in summer, while Lake Erhai showed peak levels in winter. Throughout both seasons, intracellular MCs dominated over extracellular MCs, with MC-LR serving as the primary congener (Figure 4). Ecological risk assessments indicated that all lakes were within the safe range (RQ_f_ = 0), except for Lake Erhai in winter, which posed a high risk (RQ_f_ = 1.13) due to elevated intracellular MC-LR concentrations exceeding the predicted no-effect concentration (PNEC) (Table S4).

**Figure 4:**
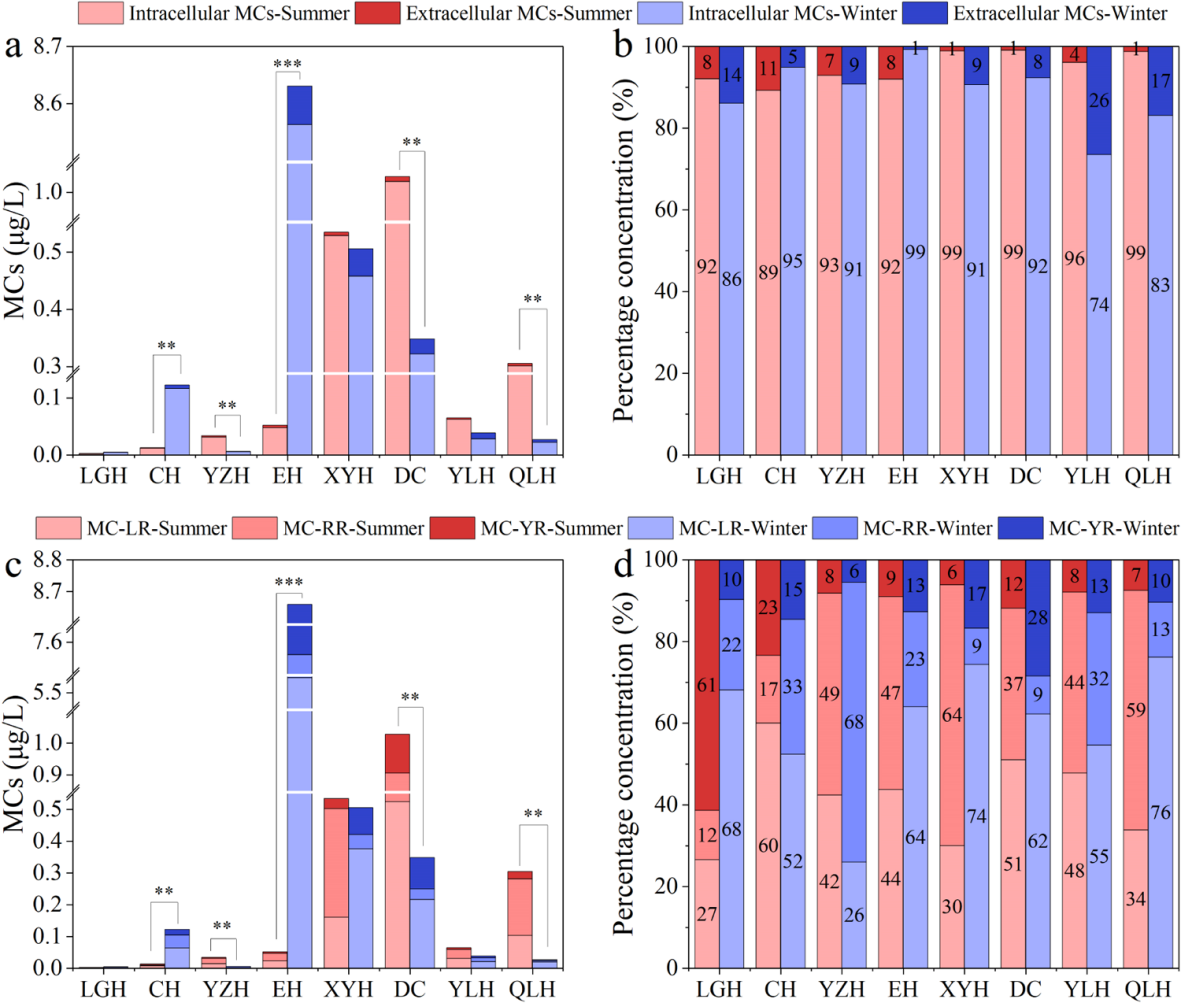
Concentrations (a, c) and percentage contributions (b, d) of various microcystins (MC) types in the eight Yunnan Plateau lakes during summer and winter. Extracellular MCs = Extracellular microcystins; Intracellular MCs = Intracellular microcystins; MC-LR = Microcystin LR; MC-YR = Microcystin YR; MC-RR = Microcystin RR. Asterisks (*) indicate significant path coefficients: *P ≤ 0.05, **P ≤ 0.01, ***P ≤ 0.001. Refer to Figure 1 for lake abbreviations.

### 3.2 Relationships Between MC Concentrations and Environmental Factors

Spearman rank correlation analyses revealed that MC concentrations in summer were significantly and positively correlated with Turb, TN, TP, Chl *a*, BCyan, DCyan, species number (SN), richness, and BCyan-MP, DCyan-MP, and DCyan-MP% (*P* ≤ 0.05), and negatively correlated with *Z*_M_ and N/P (Figure 5a). In winter, MC concentrations were positively correlated with Turb, AT*SRI, DCyan-MP, DCyan-MP%, and BCyan-MP% (Figure 5b). MC-LR followed a similar pattern to MCs, except for the absence of correlations with N/P and DCyan-MP% in summer.

**Figure 5:**
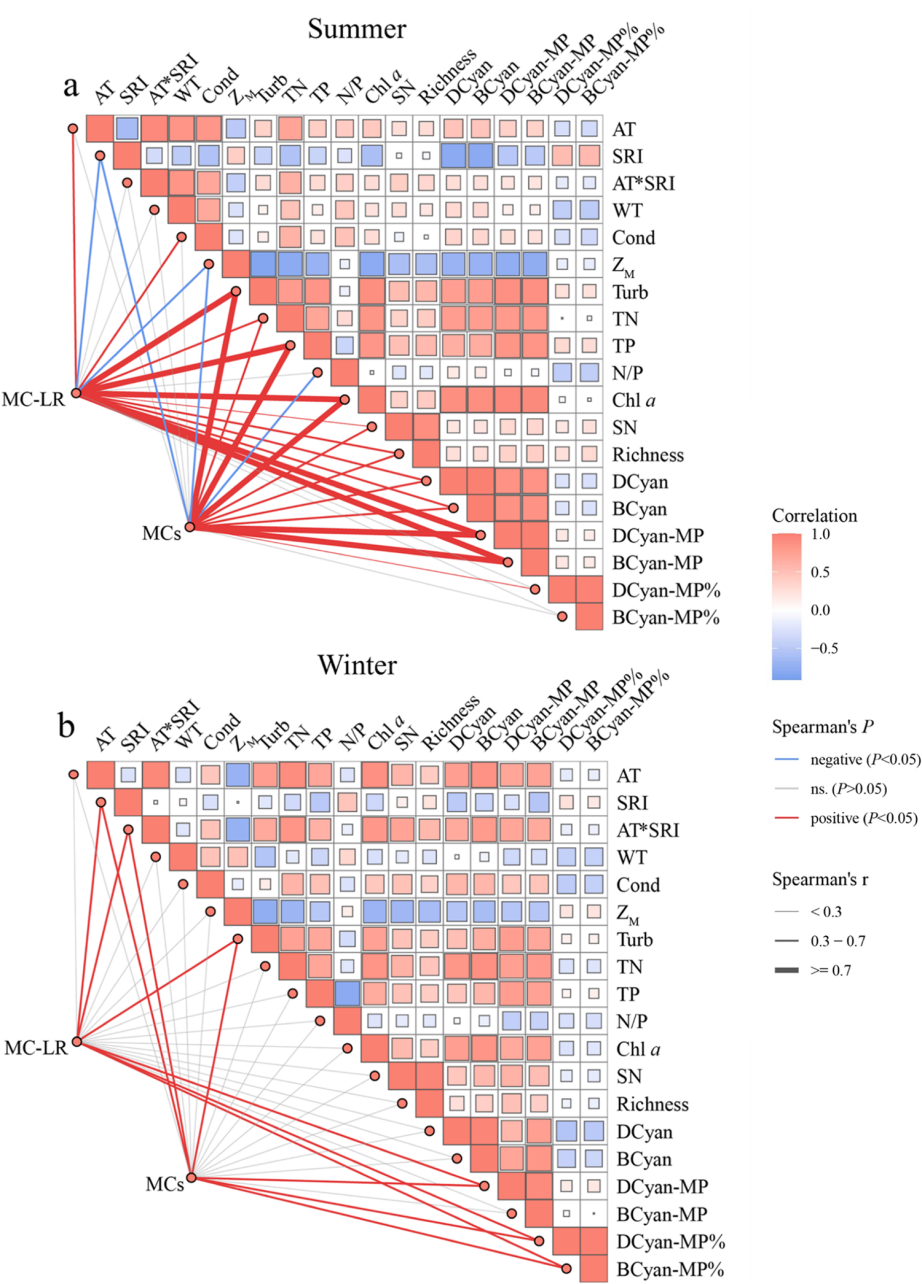
Spearman rank correlation analysis of microcystins (MCs) with physicochemical and phytoplankton factors in eight Yunnan Plateau lakes during summer (a) and winter (b). Refer to Figures 2–4 for abbreviations.

Multiple stepwise regression analysis identified SRI, BCyan-MP%, and WT as the most important environmental factors influencing MC production in summer, whereas BCyan-MP%, TP, and SRI dominated in winter (Table 1).

**Table 1:** Multiple Stepwise Regression of Microcystins on Environmental Factors in the Eight Yunnan Plateau Lakes during Summer and Winter^a^

season	variable	nonstandardized coefficient (B)	SE	standardized coefficient (β)	*t*	*P*	*F*
summer	constant	16.57	2.83		5.86	<0.001	18.98
	SRI	–0.05	0.01	–0.73	–6.17	<0.001	
	BCyan-MP%	0.52	0.12	0.45	4.23	<0.001	
	WT	–0.11	0.04	–0.38	–3.03	0.004	
winter	constant	–58.81	24.91		–2.36	0.022	10.61
	BCyan-MP%	4.27	1.34	0.40	3.20	0.002	
	TP	–18.10	7.79	–0.33	–2.33	0.024	
	SRI	0.42	0.18	0.30	2.35	0.023	

a Note: SE, the standard error. Refer to Figures 2–4 for abbreviations.

Random Forest analysis ranked TN, TP, Chl *a*, and BCyan-MP% as top summer drivers, while BCyan, TP, and SRI were most important in winter (Figure 6).

**Figure 6:**
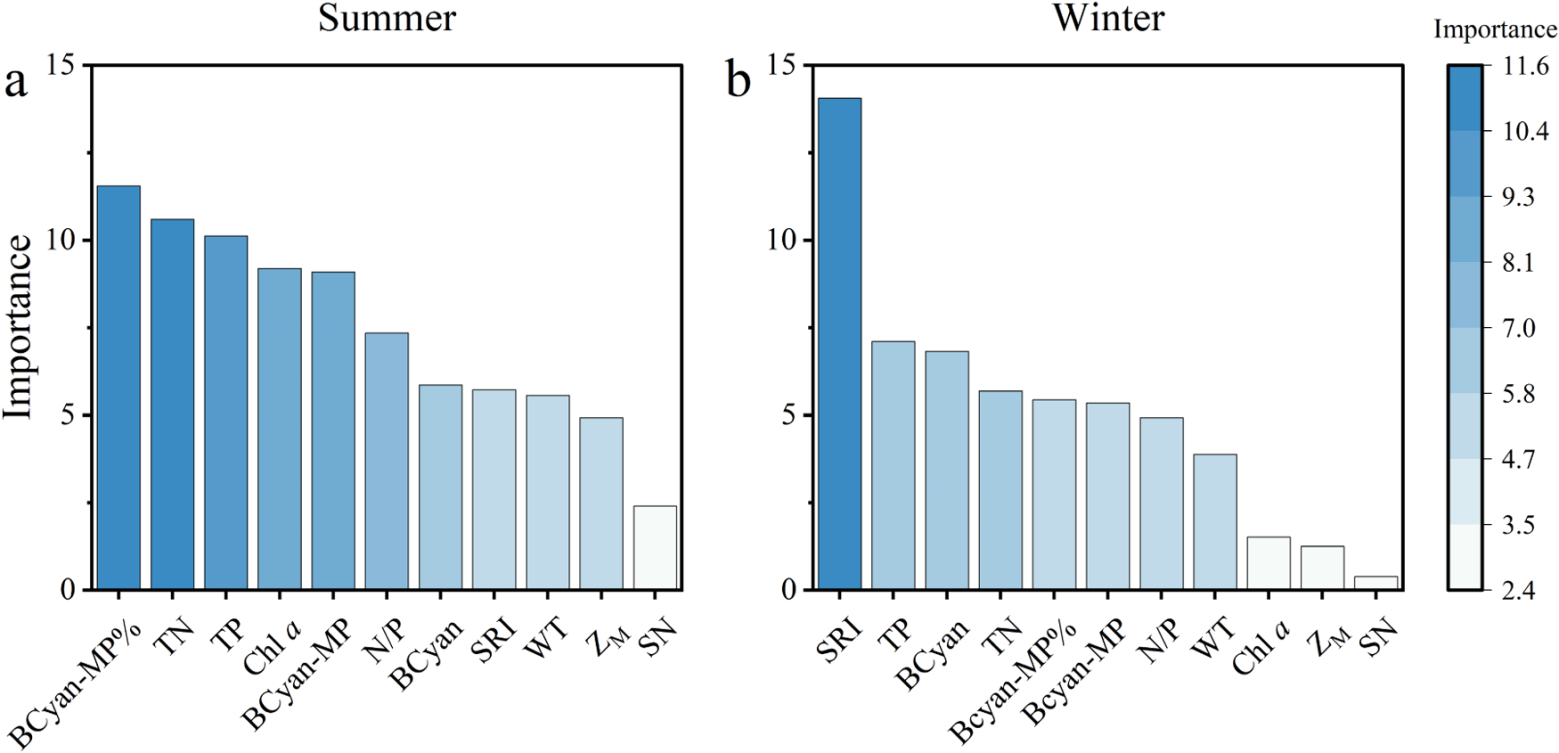
Random Forest analysis of microcystins (MCs) with physicochemical and phytoplankton factors in eight Yunnan Plateau lakes during summer (a) and winter (b). Refer to Figures 2–4 for abbreviations.

Linear regression analyses indicated that MC concentrations increased with TN, TP, Chl *a*, BCyan, DCyan, BCyan-MP, DCyan-MP, BCyan-MP%, and DCyan-MP% in summer, and with SRI, BCyan, BCyan-MP, DCyan-MP, BCyan-MP%, and DCyan-MP% in winter (Figure 7). Notably, MC concentrations in summer decreased with increasing SRI, with significant suppression observed at a solar radiation intensity of approximately 290 W/m^2^ (Figure 7a). Winter MC concentrations were elevated at low TN/TP but showed stronger responses to high nutrients in summer. Notably winter concentrations exhibited greater variability and exposure risks at similar BCyan levels. The positive correlation in winter may arise from low-temperature and light, where cyanobacteria upregulate MC production as a stress response under high-light, low-temperature conditions.

**Figure 7:**
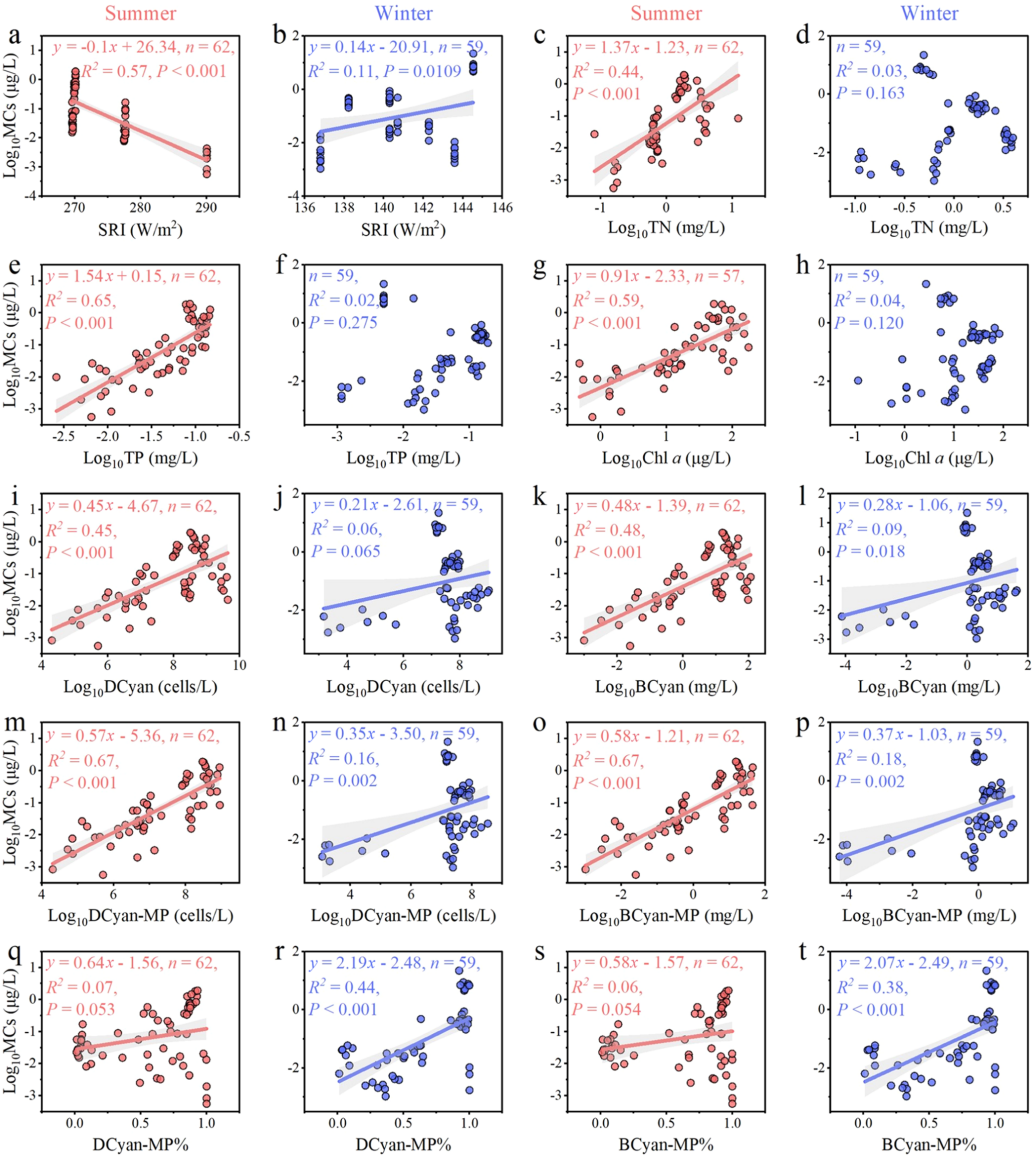
Linear regression relationships of microcystins (MCs) with physicochemical factors (a–f), and phytoplankton parameters (g–t) in the eight studied Yunnan Plateau lakes during summer (red) and winter (blue). Shaded areas represent 95% confidence intervals. Refer to Figures 2–4 for abbreviations.

Partial least-squares-structural equation modeling (PLS-SEM) showed that Cyan-MP% had the greatest effect on MC concentrations in summer (standardized total effect = 0.45), followed by algae abundance (score = 0.26). This aligns with the positive correlations observed between BCyan-MP and MC concentrations in summer (Figure 5a), confirming the direct role of toxigenic cyanobacteria in MC production. In winter, nutrients (TN, TP) exhibited the strongest negative (score = −0.77), followed by Cyan-MP% and AT*SRI (scores = 0.51 and 0.46, respectively). Notably, nutrients were negatively correlated with MC concentrations in winter (Figure 8).

**Figure 8:**
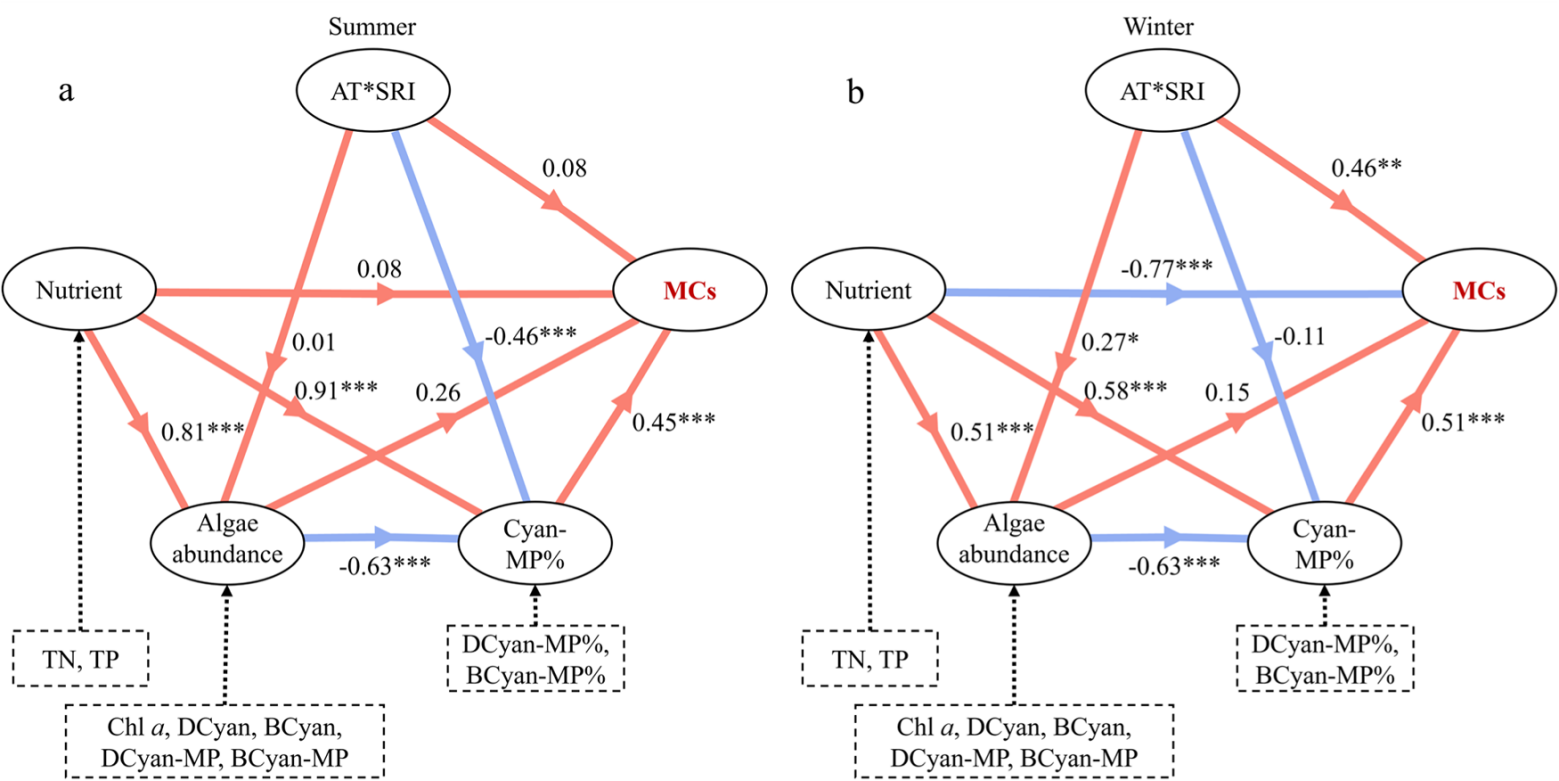
Partial least-squares-structural equation modeling between microcystins (MCs), physicochemical factors, and phytoplankton factors in eight Yunnan Plateau lakes during summer (a) and winter (b). Numbers on arrows indicate effect sizes, with red and blue arrows representing positive and negative effects, respectively. Asterisks (*) indicate significant path coefficients: *P ≤ 0.05, **P ≤ 0.01, ***P ≤ 0.001. Refer to Figures 2–4 for abbreviations.

## 4 Discussion

### 4.1 Microcystin Concentrations in the Eight Lakes

Microcystin (MC) concentrations in the eight Yunnan Plateau lakes exhibited significant spatial and temporal variations, with higher concentrations observed in lakes Dianchi, Erhai, and Xingyunhu. These findings align with previous studies on the eutrophication status of these lakes.^43^ Notably, mean MC concentrations exceeded the World Health Organization (WHO) provisional drinking water guideline of 1 μg/L in Lake Dianchi during summer (5 of 8 sampling sites) and Lake Erhai during winter (all 8 sites), indicating potential water safety risks in these regions. These results underscore the need for targeted management strategies in drinking water source lakes, such as nutrient reduction in eutrophic systems and seasonal monitoring. Although Lake Erhai is at a high level of MC ecological risk in winter, the other lakes, particularly those serving as drinking water sources, still face potential threats that cannot be ignored.

Southern-central, densely populated lakes in Yunnan (e.g., Dianchi, Xingyunhu, Qiluhu, Yilonghu, and Yangzonghai) contained higher MC concentrations in summer, likely linked to anthropogenic nutrient inputs, as evidenced by higher TN/TP levels during this season. In contrast, lakes in the less-populated northwestern regions (e.g., Chenghai, Luguhu, and Erhai) showed winter MC peaks, which may be attributed to the region’s higher light intensity. This temporal pattern aligns with findings from Zhu et al. and Yu et al.,^23,44^ implicating anthropogenic nutrient inputs may play a significant role in driving MC production in these lakes.

### 4.2 Key Environmental Factors Influencing MC Concentrations

Our analysis identified TP, TN, Turb, Chl *a*, and DCyan-MP% as the most significant factors positively correlated with MC concentrations. These findings are consistent with previous studies,^30,44^ highlighting the critical role of nutrients in driving cyanobacterial growth and MC concentrations. The positive relationship between turbidity and MCs likely reflects an indirect relationship mediated by changes in algal biomass (Chl *a*) and cyanobacterial biomass (BCyan), as observed in other eutrophic lakes in China.^45^

Interestingly, we observed a negative correlation between the total N/P and MC concentrations, particularly in lakes Dianchi, Xingyunhu, and Erhai, where the mean N/P ratio was 20.3. This finding aligns with studies in eutrophic Canadian lakes,^46^ suggesting that nutrient stoichiometry may influence MC production. Additionally, the seasonal variation in the relationship between solar radiation intensity (SRI) and MC concentrations, where MCs increased with SRI in winter but were suppressed at higher SRI levels in summer, supports the hypothesis that light intensity plays a regulatory role in MC production. This pattern is consistent with previous observations that high light intensities can shift cyanobacterial communities toward toxin-producing species and alter MC variant profiles.^47,48^

### 4.3 Interaction of Temperature and Solar Radiation

Contrary to previous studies,^30,49^ water temperature showed no significant correlation with MC concentrations in the Yunnan Plateau lakes. We hypothesize that the high solar radiation intensity in this region may override the temperature-dependent effects on cyanobacterial growth and MC production. This is supported by our observation that SRI promoted MC production at moderate intensities during winter but inhibited it at higher intensities during summer. The combined effect of air temperature and SRI on MCs indicates that photoinhibition in near-surface waters may play a significant role in regulating MC concentrations in high-altitude lakes. The mechanism of light-driven degradation of MC-LR involves the synergistic oxidative pathways. During photolysis, UV–C (254 nm) triggers isomerization (e.g., 4­(E)-6­(Z)-Adda and cyclization products) by directly exciting conjugated double bond of the Adda side chain of MC-LR (λ_max = 238 nm), significantly reducing its binding affinity^50^ to protein phosphatases­(PP1/PP2A). In contrast, VUV (185 nm) generates highly reactive −OH through water molecule cleavage, which can nonselectively attack the aromatic ring and peptide bonds of MC-LR for rapid mineralization.^51^ Under light-limiting conditions, nontoxic Microcystis strains exhibit higher competitiveness. At high light intensities, nontoxic *Microcystis* had a higher proportion of photosystem II shutdown and was more sensitive to light. Toxic *Microcystis* responded to high light stress through strong nonphotochemical quenching (qN), whereas nontoxic *Microcystis* were severely photoinhibited due to limited qN formation.^52^ These findings reveal that toxic *Microcystis* is more resistant to high light conditions in plateau, yet it experiences a faster rate of MCs degradation, i.e., exhibiting a lower concentration of MCs at the same level of Chl *a*. This is compatible with our results that reduced light availability in winter alleviates photoinhibition, thereby explaining in some lakes the higher MC concentrations in winter than in summer.

The regional comparison of MC concentrations per unit of Chl *a* revealed that Yunnan Plateau^23,44,53^ lakes generally exhibited lower MC levels compared to lakes in the Eastern Plains^45^ and Southern China.^54^ This pattern suggests that the risk of elevated MC concentrations is higher in lower-altitude and lower-latitude regions, where temperature and solar radiation intensity are most conducive to cyanobacterial growth. The unique combination of high solar radiation and moderate temperatures in the Yunnan Plateau may explain the observed suppression of MC production in these lakes.

### 4.4 Limitations and Future Directions

While our study provides valuable insights into the factors influencing MC production in high-altitude lakes, several limitations should be acknowledged. First, the granularity of solar radiation data in this study was limited to monthly averages, and future research should incorporate continuous measurements of solar radiation intensity, light quality, and photosynthetic spectral data to better understand the regulatory effects of light on MC production. Experimental studies with controlled UV radiation level would quantify MC production responses to specific light spectra. Additionally, our sampling design focused on two annual time points, which may have missed short-term dynamics. It would be valuable to do less spatially intense sample but at high resolution, i.e., weekly, which can better capture environmental drivers. Therefore, enriching the comparison of light intensity, temperature, algae and MCs in plateau and lowland areas is a future direction that could be investigated. It is necessary to set up multiscale culture experiments to verify the effects of MCs control through synergistic interactions among light intensity, nutrients and algal species.

## 5 Conclusions

This study highlights the complex interplay of environmental factors influencing MC production in Yunnan Plateau lakes. Key drivers include TP, TN, Turb, and Chl *a*, with solar radiation intensity emerging as a primary biological mediator. The higher exposure risk in winter, coupled with the negative impact of higher solar radiation on MC concentrations in summer, underscores the need for region-specific cyanobacterial management strategies. Our findings contribute to a broader understanding of the conditions regulating MC production in high-altitude lakes, demonstrating that photoinhibition associated with high solar radiation may play a significant role in suppressing cyanobacterial growth and MC production in these regions. Future research should set up multiscale culture experiments to verify the effects of MCs control through synergistic interactions among light intensity, nutrients and algal species, informing more effective mitigation strategies.

## Supplementary Material



## Notes

The characteristics of lakes, physicochemical and phytoplankton-associated characteristics, ecological risks of MCs in the eight Yunnan Plateau lakes (PDF)
