# Supplementary material for: Combined Effects of Irradiation, Nutrients, and Cyanobacterial Composition on Microcystin Concentration in Chinese Plateau Lakes
Source: Environ Health (Wash). 2025 Jul 10;3(11):1366–79. doi: 10.1021/envhealth.5c00123 (PMC12645303; doi:10.1021/envhealth.5c00123)
Supplement: Supplementary file 1 [file eh5c00123_si_001.pdf]

Supporting information

Combined effects of irradiation, nutrients, and cyanobacterial composition on microcystin concentration in Chinese Plateau lakes

Hexiang Zhao[1,†], Xu Zhao[1,12,†], Ying Liu[1 *], Yanfeng Sun[1], Youai Duan[13], Jun Chen[2], Ping Xie[1,2], Yong Liu[1], Xinyu Miao[1], Haijun Wang[1], Chi Xu[3], Qian Liu[4], Wenyu Yang[1], Christian E.W. Steinberg[5,6], Hugh MacIsaac[1,7], Susanna A. Wood [8], Erik Jeppesen[1,9,10], Hans W. Paerl [11]

[1] Yunnan Key Laboratory of Ecological Protection and Resource Utilization of River-lake Networks, Qilu Lake Field Scientific Observation and Research Station for Plateau Shallow Lake in Yunnan Province, Institute for Ecological Research and Pollution Control of Plateau Lakes, School of Ecology and Environmental Science, Yunnan University, Kunming 650500, China

[2] Donghu Experimental Station of Lake Ecosystems, State Key Laboratory of Freshwater Ecology and Biotechnology, Institute of Hydrobiology, Chinese Academy of Sciences, Wuhan 430072, China

[3] School of Life Sciences, Nanjing University, Nanjing 210023, China

[4] State Key Laboratory of Environmental Chemistry and Ecotoxicology, Research Center for Eco-Environmental Sciences, Chinese Academy of Sciences, Beijing 100085, China

[5] Institute of Biology, Freshwater & Stress Ecology, Humboldt University, Berlin 12437, Germany

[6] Yunnan Provincial Key Laboratory of Soil Carbon Sequestration and Pollution Control, Faculty of Environmental Science & Engineering, Kunming University of Science & Technology, Kunming 650500, China

[7] Lincoln University, 85084 Ellesmere Junction Road, Lincoln 7647, New Zealand

[8] Great Lakes Institute for Environmental Research, University of Windsor, Windsor, ON, N9B 3P4, Canada

[9] Department of Ecoscience, Aarhus University, Aarhus 8200, Denmark

[10] Sino-Danish Centre for Education and Research, Beijing 101408, China

[11] Institute of Marine Sciences, Department of Earth, Marine and Environmental Sciences, University of North Carolina at Chapel Hill, 3431 Arendell Street, Morehead City, NC 28557, USA

[12] Yunnan Institute of Water &Hydropower Engineering Investigation, Design and Research, Kunming 650032, China

[13] Yunnan Ecological and Environmental Monitoring Center, Kunming 650034, China

†These authors share their first authorship.

*Corresponding authors. Email: ly@ynu.edu.cn

Table S1. The characteristics of the eight Yunnan Plateau lakes.

	CH	LGH	EH	QLH	XYH	YLH	DC	YZH
Longitude (E°)	100.66	100.79	100.02	102.79	102.78	102.59	102.71	103.01
Latitude (N°)	26.54	27.72	25.78	24.17	24.34	23.67	24.83	24.91
Area (km^2)	75.97	50.10	249.00	26.95	34.71	33.87	297.90	31.00
Z_M (m)	18.08	31.29	10.76	3.22	6.88	2.22	4.82	20.79
Z_{Max} (m)	35.87	93.50	20.70	6.84	11.00	6.55	11.35	31.00
Functionality	DDW	DDW	DDW	RW	RW	RW	ADW	ADW

Note: Area, lake surface area; ZM, mean depth; ZMax, maximum depth; DDW, direct drinking water source; ADW, alternate drinking water source; RW, recreational waterbodies; LGH, Lake Luguhu; CH, Lake Chenghai; YZH, Lake Yangzonghai; EH, Lake Erhai; XYH, Lake Xingyunhu; DC, Lake Dianchi; YLH, Lake Yilonghu; QLH, Lake Qiluhu.

Table S2. The main physicochemical characteristics (mean ±s.d.) of the eight studied lakes.

Lakes	Season	CH	LGH	EH	QLH	XYH	YLH	DC	YZH
AT (℃)	Summer	21.85	14.85	20.85	21.85	21.85	22.85	20.85	20.85
	Winter	8.85	1.85	8.85	9.85	9.85	11.85	9.85	8.85
SRI (W/m^2)	Summer	277.47	290.03	277.67	269.91	269.91	269.59	270.10	269.68
	Winter	140.70	143.59	144.53	140.29	140.29	142.28	138.21	136.79
AT*SRI	Summer	6062.74	4306.93	5789.48	5897.47	5897.47	6160.02	5631.65	5622.83
	Winter	1245.16	265.64	1279.13	1381.87	1381.87	1686.03	1361.41	1210.63
WT (℃)	Summer	25.09±0.32	20.41±0.35	23.96±0.79	24.81±0.59	24.44±0.50	26.49±0.54	22.86±0.24	23.77±0.21
	Winter	17.18±0.25	11.46±2.20	13.46±1.26	12.93±0.79	12.77±0.36	13.07±0.56	11.38±0.34	15.08±0.54
DO (mg/L)	Summer	6.29±0.15	6.43±1.27	7.17±1.13	9.80±1.89	9.77±1.48	7.88±0.51	8.30±0.58	7.76±0.38
	Winter	6.54±0.18	6.80±1.88	8.58±0.33	9.58±1.51	7.36±0.32	8.69±0.77	8.52±0.43	6.64±0.28
DO (%)	Summer	76.7±1.94	71.4±14.41	85.36±14.33	118.52±23.72	116.95±18.43	98.00±6.56	95.46±6.81	91.89±4.50
	Winter	68.15±1.97	62.01±20.29	82.61±4.50	90.48±12.69	70.09±3.28	82.95±7.73	78.15±4.31	65.94±2.52
Cond (μS/cm)	Summer	1386.67±8.33	217.36±11.22	321.23±4.93	1123.17±95.16	580.33±3.14	554.5±8.83	404.36±7.24	410.88±2.70
	Winter	1103.33±6.15	151.90±17.52	227.50±8.03	685.00±8.22	431.94±7.00	416.13±6.54	288.90±5.20	355.40±2.98
pH	Summer	8.90±0.01	8.06±0.70	8.24±0.22	8.72±0.05	8.70±0.09	8.51±0.04	8.85±0.09	8.71±0.07
	Winter	8.35±0.05	7.37±0.40	7.84±0.22	7.93±0.08	7.72±0.17	7.56±0.10	7.92±0.06	7.48±0.08

Z_M (m)	Summer	18.08±7.31	31.29±27.05	10.76±3.84	3.22±1.23	6.88±2.15	2.22±1.07	4.82±0.92	20.79±5.92
	Winter	18.55±7.17	32.37±26.95	11.21±3.31	3.90±1.30	6.69±2.07	2.45±1.26	4.98±0.98	21.75±5.69
Z_{SD} (m)	Summer	5.88±1.73	4.76±3.07	1.09±0.19	0.49±0.11	0.69±0.11	0.30±0.09	0.48±0.10	1.55±0.16
	Winter	1.01±0.21	7.01±4.13	1.38±0.22	0.20±0.06	0.34±0.04	0.12±0.04	0.19±0.04	2.58±0.37
Turb (NTU)	Summer	0.75±0.18	2.87±4.14	6.22±1.6	30.72±10.96	23.57±14.85	34.65±10.69	46.10±24.81	3.19±0.79
	Winter	7.41±2.64	5.25±11.85	6.65±2.02	22.80±4.07	17.97±2.47	24.08±3.41	47.35±6.06	1.61±0.54
TN (mg/L)	Summer	0.72±0.01	0.33±0.28	0.63±0.07	5.15±3.52	1.52±0.12	3.69±0.44	1.88±0.38	0.57±0.20
	Winter	0.89±0.03	0.16±0.07	0.50±0.06	3.72±0.19	1.54±0.09	3.34±0.08	1.94±0.25	0.61±0.14
TP (mg/L)	Summer	0.01±0.00	0.01±0.01	0.05±0.03	0.12±0.01	0.13±0.01	0.08±0.02	0.10±0.02	0.02±0.00
	Winter	0.06±0.02	0.01±0.01	0.01±0.00	0.13±0.02	0.15±0.02	0.04±0.01	0.15±0.02	0.02±0.00
N/P	Summer	120.57±81.75	24.62±6.45	18.64±13.81	41.90±26.47	11.56±0.67	46.33±7.00	19.67±2.15	23.58±8.36
	Winter	17.12±4.43	79.14±79.05	29.69±0.00	29.74±4.17	10.12±0.99	88.75±11.81	13.41±2.42	37.34±11.00

Note: AT, air temperature; SRI, solar radiation intensity; AT*SRI, the product of AT and SRI, represents the combined effect of air temperature and solar radiation. WT, water temperature; DO, dissolved oxygen; Cond, conductivity; Z_M, mean depth; Z_{SD}, Secchi depth; Turb, turbidity; TN, total nitrogen; TP, total phosphorus; N/P, ratio of total nitrogen to total phosphorus; mean, the value averaged from all the sampling sites for each lake; s.d., the standard deviation. Refer to Figure 1 for explanation of lake name abbreviations.

Table S3. The main phytoplankton-associated variables (mean ± s.d.) of the eight studied lakes.

Lakes	Season	CH	LGH	EH	QLH	XYH	YLH	DC	YZH
Chl *a* (μg/L)	Summer	1.16±0.61	2.93±3.50	12.76±7.93	120.85±35.68	41.48±18.38	78.46±17.83	72.64±34.65	13.24±2.63
	Winter	8.08±2.16	2.34±3.60	6.55±2.12	39.58±4.65	37.83±19.14	51.18±6.15	38.01±19.31	10.51±3.34
SN	Summer	21.83±2.04	25.00±15.17	36.75±5.14	28.67±3.78	36.50±4.23	39.50±3.08	30.91±3.99	17.38±3.34
	Winter	18.00±2.10	13.29±6.58	16.50±3.85	32.50±4.37	25.29±2.06	23.33±3.88	17.18±3.22	11.50±2.20
DCyan (cells/L)	Summer	(2.92±4.40) $\times10^6$	(3.88±7.69) $\times10^6$	(9.61±6.43) $\times10^6$	(9.59±3.11) $\times10^8$	(1.16±0.18) $\times10^8$	(3.35±0.69) $\times10^9$	(4.95±2.11) $\times10^8$	(2.57±0.45) $\times10^8$
	Winter	(3.01±0.60) $\times10^7$	(1.16±1.95) $\times10^5$	(1.67±0.32) $\times10^7$	(2.76±1.73) $\times10^8$	(5.08±1.71) $\times10^7$	(5.85±3.25) $\times10^8$	(6.18±2.45) $\times10^7$	(6.07±1.18) $\times10^7$
BCyan (mg/L)	Summer	0.11±0.15	0.17±0.32	0.50±0.28	36.79±10.82	5.67±0.83	87.72±17.33	24.75±10.86	12.02±2.20
	Winter	1.62±0.32	0.01±0.01	0.94±0.20	8.56±4.91	2.70±1.03	22.56±14.01	3.28±1.34	1.83±0.45
Richness (d)	Summer	0.95±0.22	1.19±0.71	1.45±0.30	1.34±0.16	1.90±0.23	1.76±0.14	1.50±0.19	0.85±0.17
	Winter	0.99±0.12	0.98±0.55	0.92±0.23	1.62±0.18	1.36±0.12	1.11±0.19	0.90±0.17	0.59±0.12
Evenness (J')	Summer	0.55±0.06	0.60±0.11	0.66±0.08	0.55±0.05	0.41±0.04	0.38±0.04	0.35±0.05	0.20±0.07
	Winter	0.53±0.04	0.37±0.28	0.37±0.10	0.55±0.05	0.46±0.08	0.42±0.07	0.28±0.06	0.57±0.04
Shannon	Summer	1.45±0.25	1.66±0.56	2.11±0.36	1.85±0.14	1.47±0.19	1.39±0.16	1.21±0.18	0.56±0.17
	Winter	1.52±0.14	1.02±0.94	1.04±0.32	1.90±0.16	1.47±0.26	1.30±0.23	0.79±0.18	1.38±0.08
Simpson	Summer	0.65±0.08	0.70±0.13	0.81±0.10	0.77±0.05	0.51±0.06	0.63±0.07	0.52±0.08	0.23±0.08

	Winter	0.71±0.05	0.39±0.34	0.42±0.14	0.79±0.04	0.57±0.10	0.61±0.09	0.32±0.09	0.71±0.02
DCyan-MP (cells/L)	Summer	(4.16±5.01) $\times10^{5}$	(2.80±5.02) $\times10^{6}$	(5.98±3.12) $\times10^{6}$	(5.72±1.90) $\times10^{8}$	(1.00±0.18) $\times10^{8}$	(1.56±0.35) $\times10^{8}$	(4.41±1.93) $\times10^{8}$	(7.75±5.65) $\times10^{6}$
	Winter	(1.87±0.40) $\times10^{7}$	(2.96±0.51) $\times10^{5}$	(1.61±0.32) $\times10^{7}$	(1.45±1.04) $\times10^{8}$	(4.71±1.65) $\times10^{7}$	(4.41±0.38) $\times10^{8}$	(1.87±2.38) $\times10^{7}$	(1.87±0.42) $\times10^{7}$
BCyan-MP (mg/L)	Summer	0.021±0.025	0.152±0.273	0.390±0.199	28.882±9.646	5.229±0.820	8.025±1.851	23.137±10.171	0.430±0.306
	Winter	1.301±0.269	0.002±0.003	0.914±0.216	5.783±3.821	2.508±1.006	1.522±1.121	3.156±1.295	0.620±0.125
DCyan-MP%	Summer	0.32±0.30	0.80±0.21	0.68±0.21	0.60±0.06	0.86±0.02	0.05±0.01	0.89±0.03	0.03±0.03
	Winter	0.62±0.03	0.56±0.43	0.96±0.03	0.51±0.09	0.93±0.03	0.07±0.03	0.96±0.03	0.36±0.05
BCyan-MP%	Summer	0.37±0.32	0.91±0.12	0.81±0.15	0.78±0.05	0.92±0.01	0.09±0.02	0.94±0.02	0.04±0.03
	Winter	0.80±0.05	0.65±0.41	0.97±0.02	0.65±0.09	0.93±0.03	0.07±0.03	0.96±0.02	0.35±0.06

Note: Chl *a*, phytoplankton chlorophyll *a*; SN, species number; DCyan, cyanobacteria cell density; BCyan, cyanobacteria biomass; Richness, species richness index; Evenness, species evenness index; DCyan-MP, density of microcystin-producing cyanobacteria; BCyan-MP, biomass of microcystin-producing cyanobacteria; DCyan-MP %, percentage of DCyan-MP to DCyan; BCyan-MP %, percentage of BCyan-MP to BCyan; mean, the value averaged from all the sampling sites for each lake; s.d., the standard deviation. Refer to Figure 1 for explanation of lake name abbreviations.

Table S4. Ecological risks of MCs in the eight Yunnan Plateau lakes.

Lake	Season	MEC	NO_1	NO_2	RQ	F	RQ_f
CH	Summer	0.013	0	6	0.0023	0	0
LGH	Summer	0.002	0	7	0.0004	0	0
EH	Summer	0.052	0	12	0.0091	0	0
QLH	Summer	0.305	0	6	0.0532	0	0
XYH	Summer	0.535	0	6	0.0934	0	0
YLH	Summer	0.065	0	6	0.0113	0	0
DC	Summer	1.027	0	11	0.1793	0	0
YZH	Summer	0.034	0	8	0.0059	0	0
CH	Winter	0.112	0	6	0.0213	0	0
LGH	Winter	0.005	0	7	0.0009	0	0
EH	Winter	8.63	6	8	1.5062	0.75	1.13
QLH	Winter	0.027	0	6	0.0047	0	0
XYH	Winter	0.506	0	7	0.0883	0	0
YLH	Winter	0.039	0	6	0.0067	0	0
DC	Winter	0.348	0	11	0.0608	0	0
YZH	Winter	0.006	0	8	0.0011	0	0

Note: MEC, measured environmental concentration of MCs; NO_1, number of samples larger than PNEC, PNEC is the predicted no-effect concentration of MCs (5.73 μg/L); NO_2, total sample number; RQ, risk quotient; F, frequency of MEC over PNEC; RQ_f, optimized risk quotient; $RQ_f > 1$ is high risk.
